# Prolonged Screen Exposure During COVID-19—The Brain Development and Well-Being Concerns of Our Younger Generation

**DOI:** 10.3389/fpubh.2021.700401

**Published:** 2021-09-06

**Authors:** Agnes S. K. Wong

**Affiliations:** Ontario Institute for Studies in Education, University of Toronto, Toronto, ON, Canada

**Keywords:** public health, children, brain development, well-being, online learning, COVID-19

## Abstract

COVID-19 is a significant public health crisis and it has given a major impact especially in the field of education. The situation has forced educators around the world to shift to an online mode of teaching and children are forced to study online at home. The benefits of online learning are undeniable, but the possible long-term developmental risks of prolonged screen use should not be overlooked. Recent research findings have clearly suggested the negative effects of screen time on the brain development and well-being of our younger generation. Considering the possible long-term developmental risks of prolonged screen use, policy makers should consider appropriate public health policy (e.g., recommendations on screen time) and guideline for the implementations of online learning (e.g., allowing flexibility to suit individual needs). Multidisciplinary collaboration between policy makers, health care professionals, schools, and parents is required to rethink the current situation before it is too late.

## Introduction

COVID-19 is a significant public health crisis and has changed our lives remarkedly. In particular, most children are affected as COVID-19 prevents them to get access to face-to-face education at school. This situation has forced educators around the world to shift to an online mode of teaching and children are forced to study online at home. According to a recent study in Canada, most children have increased screen time and decreased physical activities during the pandemic (1). The strengths and opportunities of online learning are undeniable. For example, it allows time and location flexibility to cater to wide audience of any age (2). Furthermore, online learning can be considered as a great relief for parents as it helps their children continue their education online and keeps them busy (3). It is foreseeable that different online applications will be increasingly common and widely used in the field of education in the near future (4). Despite the many benefits of online learning, how might prolonged screen exposure affect the brain development and well-being of our younger generation? Furthermore, it is time to reflect on the suitability of some implementations of online learning in children.

## Prolonged Screen Exposure and Brain Development

Considering the developmental and health concerns of screen-based media use, the American Academy of Pediatrics in 2016 recommended limiting screen use for children older than 2 years to no more than 1 h per day (5). However, when children are forced to stay at home during the pandemic, the screen time of most children has increased markedly. As the brain networks of children are rapidly developing, the current situation requires our consideration. Previous studies have demonstrated that increased screen time is associated with inattention and self-regulation problems among preschool children (6, 7). In particular, a recent cross-sectional study which adopted diffusion tensor imaging (DTI) has found an inverse association between screen-based media use and microstructural integrity of brain white matter tracts (that support language, executive functions, and emergent literacy skills) in children aged 3–5 (8). Recent longitudinal studies have provided evidence on directions of effect: with screen time predicting more negative child outcomes over time, such as executive function, even after controlling for covariates such as verbal ability (9). One study with a particularly strong design (because it assessed change over three time points) revealed that increased screen time levels at age 2 and 3 were significantly related to poorer performance on developmental screening tests at age 3 and 5, respectively (10). The above studies suggested the negative effects of screen time on brain development and raised questions about the use of online learning particularly for young children.

## Prolonged Screen Exposure and Well-Being Concern

Sleep problems and onset of myopia are some of the other negative outcomes related to increased screen use and sedentary behaviors. Blue light exposure in the evening suppresses the production of melatonin, which affects sleep initiation and reduces sleep duration (11, 12). A recent systematic review and meta-analysis study has confirmed that screen time is associated with poorer sleep outcomes in infants and toddlers (13). Although higher quality designs are necessary, it is worth considering a limitation on screen time in the evening (13). Sleep problems may affect children with special needs in particular. For example, recent studies have confirmed that a significant proportion of the Autism Spectrum Disorder (ASD) population experience sleep difficulties (14, 15). Disruption in melatonin regulation is one of the reasons for sleep problems in ASD (15). Increased screen time during the pandemic may further worsen the situation as the association between media use and sleep is much more pronounced among children with ASD than those with ADHD or typical development (16). Considering the close relationship between autistic symptoms and sleep problems in children with ASD in home confinement during the pandemic (17), increased screen time may not only exacerbate the sleep problems of children with ASD, but may also increase their autistic symptoms, thereby posing detrimental consequences on the well-being of their parents. Increased screen time is also associated with the onset and progression of myopia (18). A recent large scaled study revealed a significant myopic shift among children aged 6–8 years after home confinement during COVID-19, and the prevalence of myopia increased 1.4–3 times in 2020 as compared to the previous 5 years (19). While the COVID-19 will eventually be under control and schools will resume, the increased dependence on digital devices, and the possible long-term detrimental consequences on the brain development and well-being of our younger generation deserve our attention.

## Discussions

Ever since the COVID-19, there is a sharp increasing trend in the use of different online applications such as video conferencing software, and it is foreseeable that such online applications will be increasingly common and widely used in the field of education in the near future (4). On the other hand, recent research findings have clearly suggested the negative effects of screen time on the brain development and well-being of our younger generation. This situation should be carefully addressed and reconsidered. Raising awareness among different parties, including policy makers, health care professionals, schools, and parents is the key to mitigating the situation. We should reflect on the suitability of the implementations of online learning in preschool children. During the pandemic, some typically developing preschool children are required to spend long hours to complete online learning assignments (1), while some children with special needs are required to attend prolonged online training sessions. This situation does not only pose a serious threat to the well-being of children, but also to the parents who need to manage their heavy work duties while working from home as well as managing their parenting role and homeschooling their children during the pandemic (1).

Some preschool rehabilitation services at NGOs in some countries are required to provide at least 30 min interactive video-based training sessions for children under 6 years with special needs in order to receive the usual amount of subsidy from the government. Early and prompt rehabilitation is always preferred, but we should rethink the optimal delivery of services. Instead of putting the focus on the duration of online learning and quantity of online learning assignments, being flexible to suit the individual needs is essential. For example, for children who require more supports, parents may share short videos with the health care professionals and educators about children's behaviors at home such that advice and suggestions can be provided (20). Furthermore, health care professionals and educators may discuss with parents and offer different forms of tailored trainings (e.g., phone consultation, synchronous video-based training, and asynchronous pre-recorded video clips) flexibly to suit individual needs. As different children have different needs, the amount of funding from the government should not solely depend on the duration of the video-based training session. It is crucial for different parties to work together and find what works best for the children.

## Conclusion

COVID-19 is a significant public health crisis and it has given a major impact especially in the field of education. Online learning is an important alternative to reduce the impact of school closures during the pandemic, and it is foreseeable that online learning will be increasingly common in the near future. Considering the possible long-term developmental risks of prolonged screen use, the author would like to recommend policy makers consider appropriate public health policy (e.g., recommendations on screen time) and guideline for the implementations of online learning (e.g., allowing flexibility to suit individual needs). With reference to the literature, appropriate policy and guideline for the implementations of online learning is particularly crucial for preschool education field when young children undergo rapid brain development. Multidisciplinary collaboration between policy makers, health care professionals, schools, and parents is required to rethink the current situation and provide a better learning environment for our younger generation before it is too late. It is a unique point in history and no one can be sure about the long term effects of increased screen exposure and dependence on digital devices due to the pandemic. However, without appropriate actions now, it is reasonable to speculate that multiple developmental risks (such as poorer brain development and earlier myopia onset) may appear in our next generation.

## Data Availability Statement

The original contributions presented in the study are included in the article/supplementary material, further inquiries can be directed to the corresponding author/s.

## Author Contributions

AW contributed to the whole manuscript.

## Conflict of Interest

The author declares that the research was conducted in the absence of any commercial or financial relationships that could be construed as a potential conflict of interest.

## Publisher's Note

All claims expressed in this article are solely those of the authors and do not necessarily represent those of their affiliated organizations, or those of the publisher, the editors and the reviewers. Any product that may be evaluated in this article, or claim that may be made by its manufacturer, is not guaranteed or endorsed by the publisher.
